# The effects of low-repetition and light-load power training on bone mineral density in postmenopausal women with sarcopenia: a pilot study

**DOI:** 10.1186/s12877-017-0490-8

**Published:** 2017-05-02

**Authors:** Kanako Hamaguchi, Toshiyuki Kurihara, Masahiro Fujimoto, Motoyuki Iemitsu, Koji Sato, Takafumi Hamaoka, Kiyoshi Sanada

**Affiliations:** 1 College of Sport and Health Science, Ritsumeikan University, Shiga, Japan; 2 Graduate School of Human Development and Environment, Kobe University, Kobe, Japan; 3 Department of Sports Medicine for Health Promotion, Tokyo Medical University, Tokyo, Japan

**Keywords:** Power training, Bone mineral density, Sarcopenia, Postmenopausal women

## Abstract

**Background:**

Age-related reduction in bone mineral density (BMD) is generally accelerated in women after menopause, and could be even more pronounced in individuals with sarcopenia. Light-load power training with a low number of repetitions would increase BMD, significantly reducing bone loss in individuals at risk of osteoporosis. This study investigated the effects of low-repetition, light-load power training on BMD in Japanese postmenopausal women with sarcopenia.

**Methods:**

The training group (*n* = 7) followed a progressive power training protocol that increased the load with a weighted vest, for two sessions per week, over the course of 6 weeks. The training exercise comprised five kinds of exercises (squats, front lunges, side lunges, calf raises, and toe raises), and each exercise contained eight sets of three repetitions with a 15-s rest between each set. The control group (*n* = 8) did not undergo any training intervention. We measured BMD, muscle strength, and anthropometric data.

**Results:**

Within-group changes in pelvis BMD and knee extensor strength were significantly greater in the training group than the control group (*p* = 0.029 and 0.030 for pelvis BMD and knee extensor strength, respectively). After low-repetition, light-load power training, we noted improvements in pelvis BMD (1.6%) and knee extensor strength (15.5%). No significant within- or between-group differences were observed for anthropometric data or forearm BMD.

**Conclusions:**

Six weeks of low-repetition, light-load power training improved pelvis BMD and knee extensor strength in postmenopausal women with sarcopenia. Since this training program does not require high-load exercise and is therefore easily implementable as daily exercise, it could be an effective form of exercise for sedentary adults at risk for osteoporosis who are fearful of heavy loads and/or training that could cause fatigue.

**Trial Registration:**

This trial was registered with the University Hospital Medical Information Network on 31 October 2016 (UMIN000024651).

## Background

Osteoporosis, a chronic disease involving reduced bone mineral density (BMD) [1], is one of the most prevalent factors contributing to fractures among elderly individuals [1, 2]. Age-related reduction in BMD is generally accelerated in women after menopause [3]. In addition, sarcopenia, characterized by age-related muscle atrophy [4] and weakness [5], is a primary factor influencing muscle performance [6] and is associated with an increased risk of osteoporosis [7]. An effective exercise training program to preserve BMD is needed for postmenopausal women with sarcopenia.

High-velocity resistance training, also referred to as power training, is reportedly more effective than conventional strength training for preventing osteoporosis [8, 9]. Stengel et al. [8] found that 1 year of periodised power training, designed such that 12 weeks of high-load training (70–90% 1 RM) was interleaved by 4–5 weeks of low-load training (50% 1 RM), was more effective in reducing bone loss in postmenopausal women than strength training [8]. Another study found that high-load and low-repetition power training (i.e., four sets of 3–5 repetitions with fast lifts at 85–90% of 1 RM) was effective in preserving BMD in postmenopausal women with osteoporosis or osteopenia [9]. Thus, power training appears to be more effective than strength training in reducing bone loss in individuals at risk for osteoporosis.

Although these previous studies employed high-load power training, some studies have found that maximal power output is achieved at 30–70% of 1 RM [10–12], suggesting that similar power output can be generated with a lighter load. A previous study found that peak power during light-load (35% of 1 RM) power training was comparable to or even larger than that of high-load (70% of 1 RM) power training [11]. It seems that even though the generated force output was smaller, the lighter load allowed subjects to move more quickly, resulting in greater power output. Light-load power training with a weighted vest in older postmenopausal women has been shown to increase hip BMD, demonstrating its effectiveness in preventing significant bone loss [13]. Therefore, with regard to preventing bone loss, light-load power training would likely serve as a better form of exercise training than high-load power training.

In addition to load conditions, the number of repetitions also affects muscle power output. Maximizing muscle power output throughout the training session is critical for bone adaptation, for which sufficient rest intervals between sets are required. When the total volume of exercise and rest interval between sets is equal, a low-repetition protocol provides more rest intervals than a high-repetition protocol, thereby restoring one’s ability to generate muscle power [14]. Taken together, low-repetition and light-load power training would increase BMD, reducing significant bone loss in postmenopausal women with sarcopenia who are at risk for osteoporosis. However, to our knowledge, no study has investigated the effects of low-repetition, light-load power training on BMD in these women.

Against this backdrop, the objective of this study was to investigate the effects of low-repetition, light-load power training on BMD in Japanese postmenopausal women with sarcopenia. Subjects were allocated to either the 6-week training group or control group. We hypothesized that the training group would exhibit greater increases in BMD than the control group.

## Methods

### Subjects

This study recruited middle-aged postmenopausal women with sarcopenia from local communities to perform low-repetition, light-load power training. Women who were younger than 65 years of age and at least 2 years past menopause were included. None of the subjects had cardiovascular, metabolic, or orthopaedic disease, on-going medication, unstable medical conditions, or had experienced a fracture in the past 6 months. Those who were engaged in regular exercise sessions at the time of the study were also excluded. Skeletal muscle mass index (SMI) was determined by DXA scan, and individuals with SMI < 6.12 kg/ m^−2^ (the reference value for classifying sarcopenia class 1 for Japanese women based on EWGSOP criteria [5, 15]), were diagnosed with sarcopenia and included in the study. In total, 19 postmenopausal women with sarcopenia were included. All subjects were informed of the benefits and risks of the investigation prior to providing written informed consent. This study was approved by the Ethics Committee of Ritsumeikan University (Approval number: BKC-IRB-2012-032). All the participants reviewed and signed an informed consent form, in accordance with the Declaration of Helsinki.

### Study design and intervention

**Fig. 1:**
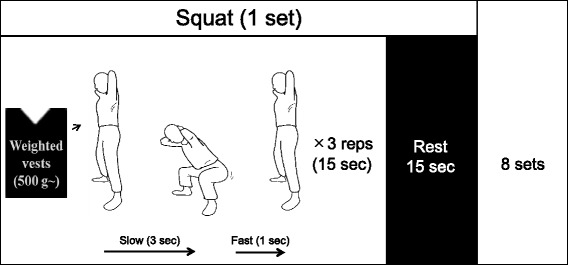
Illustration of the low-repetition and light-load power training in this study. Similar sequences were performed with four other exercises (front lunge, side lunge, calf raise, toe raise). The training group followed a progressive power training protocol that increased intensity with a weighted vest over 6 weeks, comprising 2 sessions each week

Subjects were initially allocated to either the training group or control group following a 1:1 ratio. Two subjects allocated to the training group declined to participate in exercise sessions and therefore were treated as dropouts, which resulted in eight and nine subjects in the training and control groups, respectively. The training program comprised five exercises (squats, front lunges, side lunges, calf raises, and toe raises). To accentuate power production, the concentric phase was performed as fast as possible while still maintaining the proper posture (Fig. 1). For each exercise, subjects were asked to perform eight sets of three repetitions with a 15-s rest between each set to restore mechanosensitivity of bone cells and enhance the osteogenic effect of exercise [16]. Each exercise session lasted approximately 60 min, including the warm-up and cool-down portions, which consisted of stretching and cycling exercises. The training group followed a progressive power training protocol with a weighted vest that increased in intensity over 6 weeks, comprising two sessions each week. During the first session of the power training program, subjects wore the vest without any weight in order to familiarize themselves with the training procedures. Perceived exertion was measured by the Borg Scale after each exercise to determine whether weight could be added or not; if the subject was capable of completing all eight sets, the weight was increased by 380–760 g in the next session. The weight was reduced or kept the same in the subsequent session if the subject’s perceived exertion exceeded 13 on the Borg scale (“somewhat hard” level) or if the subject complained of any muscle pain. All training sessions were conducted at the laboratory under direct supervision. Control subjects were instructed to maintain their daily physical activity level.

### Measurements

BMD (arm, spine, pelvis, leg, and total body), SMI, and muscle strength were measured at baseline and after the 6-week intervention (hereafter, Pre and Post, respectively). Subjects were given 3–7 days of rest before the post-training assessment. Dietary intake was also measured using the brief-type self-report diet history questionnaire (BDHQ).

### DXA scan

BMD, SMI, and body composition were assessed by DXA with EnCore software (Lunar Prodigy, GE Healthcare, UK). Subjects fasted overnight and did not perform any exercise in the morning before measurements. Total body and regional BMD, bone mineral content (BMC), fat mass, and fat-free mass (FFM) were analysed. All scans were performed and analysed by a single trained and licensed technician who was blinded to the group allocation. SMI was calculated as follows:

$$ \mathrm{SMI}\ \left(\mathrm{kg}/{\mathrm{m}}^2\right)=\mathrm{appendicular}\ \mathrm{skeletal}\ \mathrm{muscle}\ \mathrm{mass}\ \left(\mathrm{FFM}-\mathrm{BMC}\right)/{\left(\mathrm{body}\ \mathrm{height}\right)}^2 $$

### Muscle strength

Maximum isometric strength of the knee extensor (KE strength) on the right side was measured using a dynamometer (Biodex System 4, Sakai Med, Japan). Subjects were seated with knees flexed at 90° and hips flexed at approximately 80° [17]. Maximum voluntary contraction was performed twice, for 5 s each, with 1 min of rest between them. The maximum value was regarded as isometric muscle strength. Strength was recorded in Newton•meters (Nm) and normalized by body mass (Nm/kg). Hand grip strength (grip strength) was also assessed for each hand by a hand grip dynamometer.

### Statistical analysis

Outcome measures were BMD (arm, spine, pelvis, leg, and total body), SMI, and muscle strength. Potential differences between the control and training groups at baseline were assessed by independent *t*-tests. A two-way, mixed-design ANOVA, with group (control and training) and time (pre and post) as between- and within-subject factors, was performed to determine main and interaction effects on outcome measures. When significant interactions were identified, the Bonferroni post-hoc test was performed to detect the sources of significant differences. Within-group changes (% change) between baseline (Pre) and follow-up (Post) in BMD and muscle strength were calculated and an independent *t*-test was performed to examine if relative changes from baseline to follow-up were significantly different between the control and training groups. Within-group change was calculated as follows:

$$ \%\mathrm{change}=\left(\mathrm{Post}{\textstyle \hbox{-}}\mathrm{value}-\mathrm{Pre}{\textstyle \hbox{-}}\mathrm{value}\right)/\mathrm{Pre}{\textstyle \hbox{-}}\mathrm{value}\times 100 $$

All analyses were performed using IBM SPSS (version 19). Statistical significance was set at α < 0.05.

## Results

### Subject characteristics, attendance, and training

Of the initial 17 subjects, 15 completed all baseline and follow-up testing; one subject each from the training and control groups dropped out before follow-up testing for personal reasons unrelated to the study. Baseline characteristics of subjects in the training (*n* = 7, mean age = 60.4 ± 2.7 years) and control (*n* = 8, mean age = 60.6 ± 2.4 years) groups are presented in Table 1. There were no significant differences between groups in age, postmenopausal period, BMI, or nutritional intake (*p* > 0.05). Subjects in the training group demonstrated a high level of adherence to the exercise intervention with a participation rate above 92%, with the exception of the one subject who dropped out during the intervention. No adverse events, such as falls, fractures, or bodily pain, were reported during the 6-week intervention. The final session of power training was conducted in a manner such that perceived exertion scores were between 10 and 13 on the Borg Scale (RPE: 11.4 ± 0.8, added weights: 3.5 ± 0.8 kg).

**Table 1:** Subject characteristics

	Training (*n* = 7)	Control (*n* = 8)	*P*
Age (yrs)	60.4 ± 2.7	60.6 ± 2.3	.86
Postmenopausal period (yrs)	11.1 ± 6.3	8.9 ± 4.1	.43
Height (cm)	157.0 ± 4.3	157.6 ± 5.0	.82
Weight (kg)	47.4 ± 5.0	48.8 ± 4.4	.60
BMI (kg/m^2^)	19.2 ± 1.2	19.7 ± 1.8	.58
SMI (kg/m^2^)	5.4 ± 0.3	5.6 ± 0.5	.52
SBP (mmHg)	129 ± 26	127 ± 18	.88
DBP (mmHg)	71 ± 9	75 ± 13	.52
Energy intake (kcal/day)	1646 ± 209	1800 ± 249	.22
Protein intake (g/day)	76 ± 12	77 ± 13	.92
Calcium intake (mg/day)	661 ± 148	764 ± 194	.27
Vitamin D intake (μg/day)	22 ± 7	20 ± 8	.68
Vitamin K intake (μg/day)	406 ± 180	448 ± 237	.71

Data are presented as mean ± SD

*BMI* body mass index, *SMI* skeletal muscle mass index

*SBP* systolic blood pressure, *DBP* diastolic blood pressure

### Bone mineral density (BMD)

**Fig. 2:**
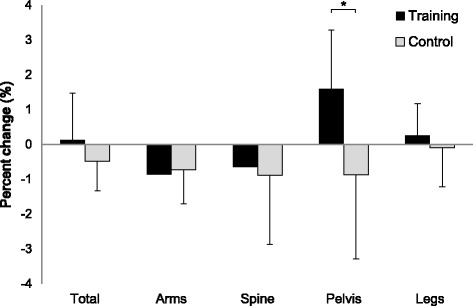
Percent change in bone mineral density after 6 weeks of training. * Significant difference between groups, *p* < 0.05

Table 2 shows baseline (Pre) and follow-up (Post) values for BMD. Corresponding F and *P* values of the time × group interaction are shown. An interaction was found for pelvis BMD, and at follow-up, smaller and larger values were found in the control and training groups, respectively (0.9% decrease and 1.6% increase, Fig. 2), with marginal significance (*p* = 0.189 and 0.059, respectively). Accordingly, the within-group change in pelvis BMD was significantly greater in the training group as compared to the control group (*p* = 0.042). No significant differences were found in within-group changes in total body BMD or BMD for other segments.

**Table 2:** Changes (mean ± SD) in bone mineral density (BMD)

	Training (*n* = 7)		Control (*n* = 8)		ANOVA Interaction	
	Pre	Post	Pre	Post	F	*P*
BMD (g/cm^2^)						
Total body	0.981 ± 0.074	0.981 ± 0.063	0.996 ± 0.057	0.992 ± 0.061	0.860	0.371
Arm	0.681 ± 0.042	0.676 ± 0.047	0.693 ± 0.042	0.688 ± 0.047	0.035	0.854
Spine	0.890 ± 0.069	0.883 ± 0.051	0.877 ± 0.077	0.870 ± 0.079	0.003	0.957
Pelvis	0.920 ± 0.076	0.935 ± 0.080	0.926 ± 0.072	0.917 ± 0.057	6.061	0.029*
Leg	0.985 ± 0.090	0.987 ± 0.082	1.026 ± 0.069	1.025 ± 0.077	0.213	0.652

*p < 0.05

### Muscle strength and SMI

**Fig. 3:**
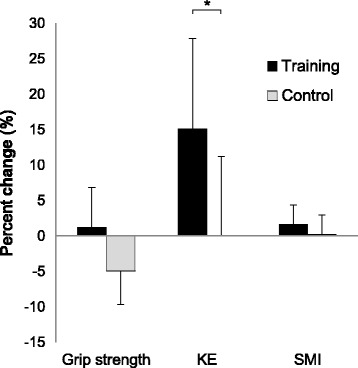
Percent change in muscle strength and skeletal muscle mass index after 6 weeks of training. KE Knee-Extensor; SMI Skeletal muscle mass index. * Significant difference between groups, *p* < 0.05

A significant group × time interaction was found for KE strength (Table 3), and a significantly larger value at follow-up (*p* = 0.030) was found in the training group (15.5% increase, Fig. 3). Accordingly, the within-group change in the training group was also significantly greater than that in the control group (*p* = 0.030). No significant main or interaction effects were found in grip strength or SMI.

**Table 3:** Changes (mean ± SD) in strength parameters

	Training (*n* = 7)		Control (*n* = 8)		ANOVA Interaction	
	Pre	Post	Pre	Post	F	*P*
Grip strength (kg)	24.3 ± 3.1	24.4 ± 3.1	25.7 ± 3.9	24.4 ± 4.3	2.748	0.121
KE strength (N/kg^2^)	1.8 ± 0.3	2.0 ± 0.3	2.0 ± 0.3	2.0 ± 0.4	5.956	0.030*
SMI (kg/m^2^)	5.4 ± 0.3	5.5 ± 0.3	5.6 ± 0.5	5.6 ± 0.4	1.044	0.326

*KE* Knee-Extensor*, SMI* Skeletal muscle mass index

*p < 0.05

## Discussion

We assessed the effects of 6 weeks of low-repetition, light-load power training on BMD, SMI, and muscle strength in postmenopausal women with sarcopenia, and found that within-group changes in pelvis BMD and KE strength were significantly greater in the training group than in the control group. This suggests that the training significantly increased pelvis BMD and KE strength.

In contrast to previous power training studies reporting that regional BMD was maintained, not increased, at follow-up in early postmenopausal women [8, 9], we found a significant within-group increase in pelvis BMD in the training group relative to the control group. The previous studies used high-load power training, while we used light-load power training, which appeared to affect peak power output during training. Peak power during light-load power training was reportedly comparable or even larger than that of high-load power training [11]. Such a difference in peak power output may have led to the variation observed in BMD outcomes. Dynamic loading is required to stimulate appositional bone growth [18], and bone adaptation is known to be influenced by peak loading magnitude [18] and velocity of load [19, 20], suggesting that the most important factor for bone adaptation is peak power. Light-load power training may have helped our subjects generate greater power output than that generated by high-load power training, thereby facilitating an increase in BMD.

Performing low-repetition exercises with 15-s rest intervals may have also contributed to increases in BMD. Low-repetition protocols provide more rest intervals than high-repetition protocols, restoring the ability to generate muscle power [14] and allowing subjects to maximize muscle power throughout the training sessions. In fact, a previous study demonstrated that power training with a longer inter-set rest period was more effective for increasing power [21]. It has also been reported that a rest interval of at least 14 s is needed to restore the mechanosensitivity of bone cells and to enhance the osteogenic effects of exercise [16]. Considered collectively, low-repetition, light-load power training would allow individuals to have sufficient rest intervals required for maximizing power output and restoring mechanosensitivity of bone cells, thereby positively affecting BMD.

With the exception of pelvis BMD, no significant improvements were found in skeletal sites in the training group. Such region-specific increases in BMD may be due to the effects of aging as well as weight-bearing. Looker et al. [22] suggested that age-related loss in BMD and weight-bearing were both greatest in the pelvis compared to other sites. In fact, although not significant, reduced BMD in the pelvis was noted in the control group at follow-up as compared to baseline (0.9% decrease, *p* = 0.189). Lower-limb power training in our study required concentric and eccentric muscle contractions of lower-limb muscles. Mechanical loading resulting from such muscle activities and weight-bearing forces may have facilitated the increase in pelvis BMD.

Knee extensor strength significantly improved by 15.5% in the training group, while no significant difference was observed in SMI. This result is consistent with results from another study that found a 16% increase in leg extension strength after 10 weeks of light-load power training (20% 1RM, 8 reps × 3 sets) in elderly individuals [23]. Since a significant increase in muscle strength was observed without any increase in muscle mass, the increase is likely attributable to neural adaptations. Increases in muscle strength can be achieved without muscle hypertrophy because of neural adaptations in the early phase of training [24, 25]. Other studies have found that neural activation was greater with power training than with strength training [25, 26]. Thus, low-repetition, light-load power training could be used to improve muscle function as well as BMD in postmenopausal women with sarcopenia.

The major limitations of this study were the small sample size and short training duration of the exercise intervention. A lack of significant differences in BMD for areas other than the pelvis, as well as SMI and grip strength, may have been due to the small sample size. Furthermore, an intervention duration of 6 weeks may not be long enough to detect significant differences in BMD, considering that other studies have indicated that significant improvements in BMD require at least 3 months to become apparent [27]. The small increase in pelvis BMD (1.6%) observed after the exercise intervention could have been due to this shorter intervention period. That said, one previous study reported that inter-day reproducibility of pelvis BMD obtained from DXA was below 1.3% [28], indicating that the 1.6% increase in pelvis BMD observed in our study would represent an actual change, while the 0.9% decrease in pelvis BMD observed in the control group appeared to be within expected measurement error. Although the decrease in the control group contributed to the significant time × group interaction in pelvis BMD, the present study demonstrated that even with 6 weeks of training, a significant relative increase could be observed at least in pelvis BMD and knee extensor strength. Future studies should investigate the long-term effects of our proposed power training program with a larger sample size.

## Conclusion

This study provides the first evidence that low-repetition, light-load power training significantly increases pelvis BMD in postmenopausal women with sarcopenia. Since this training program does not require high-load exercise, high levels of adherence would be anticipated due to its ease of implementation. We conclude that low repetition, light-load power training would be an effective form of training exercise for sedentary adults who are at risk for osteoporosis and wary of heavy loads and/or fatigable training.

## Abbreviations

BMC: Bone mineral content
BMD: Bone mineral density
DXA: Dual x-ray absorptiometry
FFM: Fat-free mass
KE: Knee extensor
Nm: Newton•meters
RM: Repetition maximum
SD: Standard deviation
SMI: Skeletal muscle mass index
